# Azelastine potentiates antiasthmatic dexamethasone effect on a murine asthma model

**DOI:** 10.1002/prp2.531

**Published:** 2019-10-29

**Authors:** Carlos D. Zappia, Ariadna Soto, Gina Granja‐Galeano, Ignacio Fenoy, Natalia Fernandez, Carlos A. Davio, Carina Shayo, Carlos P. Fitzsimons, Alejandra Goldman, Federico Monczor

**Affiliations:** ^1^ Facultad de Farmacia y Bioquímica Universidad de Buenos Aires Buenos Aires Argentina; ^2^ Instituto de Investigaciones Farmacológicas (ININFA) CONICET – Universidad de Buenos Aires Buenos Aires Argentina; ^3^ Centro de Estudios en Salud y Medio Ambiente (CESyMA) Escuela de Ciencia y Tecnología Universidad Nacional de San Martín – CONICET Buenos Aires Argentina; ^4^ Laboratorio de Patología y Farmacología Molecular Instituto de Biología y Medicina Experimental CONICET Buenos Aires Argentina; ^5^ Center for Neuroscience Swammerdam Institute for Life Sciences Faculty of Science University of Amsterdam Amsterdam The Netherlands

**Keywords:** antihistamines, asthma, azelastine, dexamethasone, glucocorticoids, histamine

## Abstract

Glucocorticoids are among the most effective drugs to treat asthma. However, the severe adverse effects associated generate the need for its therapeutic optimization. Conversely, though histamine is undoubtedly related to asthma development, there is a lack of efficacy of antihistamines in controlling its symptoms, which prevents their clinical application. We have reported that antihistamines potentiate glucocorticoids’ responses in vitro and recent observations have indicated that the coadministration of an antihistamine and a synthetic glucocorticoid has synergistic effects on a murine model of allergic rhinitis. Here, the aim of this work is to establish if this therapeutic combination could be beneficial in a murine model of asthma. We used an allergen‐induced model of asthma (employing ovalbumin) to evaluate the effects of the synthetic glucocorticoid dexamethasone combined with the antihistamine azelastine. Our results indicate that the cotreatment with azelastine and a suboptimal dose of dexamethasone can improve allergic lung inflammation as shown by a decrease in eosinophils in bronchoalveolar lavage, fewer peribronchial and perivascular infiltrates, and mucin‐producing cells. In addition, serum levels of allergen‐specific IgE and IgG1 were also reduced, as well as the expression of lung inflammatory‐related genes IL‐4, IL‐5, Muc5AC, and Arginase I. The potentiation of dexamethasone effects by azelastine could allow to reduce the effective glucocorticoid dose needed to achieve a therapeutic effect. These findings provide first new insights into the potential benefits of glucocorticoids and antihistamines combination for the treatment of asthma and grants further research to evaluate this approach in other related inflammatory conditions.

**Abbreviations**
AZE: antihistamine azelastine
DMEM: Dulbecco’s modified Eagle’s medium
GCs: Glucocorticoids
GR: glucocorticoid receptor

## 1 INTRODUCTION

Over 300 million people worldwide suffer from asthma and it is expected that 100 million more will do it in the year 2025.^1^ Asthma prevalence is variable and growing in the last decades, ranking between 2% and 10%.^2^ It is also an important global issue due to its morbidity and mortality. Asthma is the cause of 1 in every 250 global deaths and it is associated with an estimated loss of 15 million years of productive life per year (measured as disability‐adjusted life years or DALYs).^3^

We have previously reported that antihistamines, acting on the histamine H_1_ receptor, are capable of enhancing the transcriptional activity of the glucocorticoid receptor (GR),^4^ an effect that could have clinical relevance, particularly for inflammation‐associated conditions. Importantly, the GR and the H_1_ receptor are the targets of the largest number of drugs currently approved for clinical treatment.^5^ The combination of a synthetic glucocorticoid and an antihistamine is commonly administered in allergic rhinitis and atopic dermatitis.^6^, ^7^ Furthermore, coadministration of the antihistamine azelastine and the synthetic glucocorticoid mometasone synergistically ameliorated allergic inflammation in a murine model of allergic rhinitis.^8^ Therefore, coadministration therapies could lead to the design of new strategies to treat inflammation‐associated diseases. In this sense, asthma is an inflammatory disease that represents an interesting experimental scenario to extend this concept.

Glucocorticoids (GCs) are among the most effective current therapy for asthma.^9^ However, the existence of important adverse effects as well as asthmatics patients unable to control symptoms generates the need of new therapeutic strategies.^10^ Histamine has been consistently related to the development of the disease, since its identification as a potent constrictor of the smooth‐muscle airway and its increased presence in diseased tissue.^11^ Histamine levels are augmented in the airways of asthmatic patients, increasing vascular permeability, acting as chemoattractant of eosinophils and neutrophils, modulating the immune response, overall representing a key mediator between allergen, immunoglobulin E, mast cells, and asthma.^12^ However, the use of antihistamines for the treatment of asthma is an enigma. Despite the abundance of preclinical information endorsing histamine's role in asthma, there is a lack of efficacy of antihistamines in controlling its symptoms. Several clinical studies have shown that antihistamines were unable to control some of asthma symptoms in adults at regular doses,^13^ while an improvement of the symptoms was observed at higher doses.^14^

Based on previous observations in vitro 4 and in vivo,^8^ we hypothesized that the enhancement of GR transcriptional activity by antihistamines could be used to reduce GC’s effective doses used to counteract asthma symptoms, possibly resulting in new (and safer) therapeutic strategies to treat asthma. To this aim, we used an allergen‐induced murine model of asthma, as it represents the most prevalent form of asthma (allergic asthma), ideal to study the combination of a corticoid and an antiallergic drug. In this model, we specifically selected the antihistamine azelastine (AZE) to study its coadministration with the synthetic GR agonist dexamethasone (DEX) because AZE reduced the frequency of administration of inhaled GCs in chronic bronchial asthma patients.^15^ Importantly, three formulations containing AZE and GCs have been patented to treat allergic rhinitis, highlighting the potential therapeutic application of this drug combination in some inflammation‐associated respiratory conditions that could be linked to asthma.^16^, ^17^, ^18^, ^19^

Our results show that AZE potentiates DEX‐induced GR transcriptional activity in vivo, and that the combination of both drugs results in a reduction of the effective GC concentration needed to achieve a therapeutic effect in an allergen‐induced murine model of asthma. These findings provide insight into the potential benefits of GCs and antihistamines combination for the treatment of asthma symptoms and grant further research to evaluate this approach for the treatment of other inflammatory conditions.

## 2 MATERIALS AND METHODS

### 2.1 Materials

Dulbecco's modified Eagle's medium (DMEM) medium, antibiotics, phosphate‐buffered saline (PBS), DEX, and AZE were obtained from Sigma Chemical Company. Fetal calf serum (FCS) was purchased from Natocor (Córdoba, Argentina). All other chemicals were of analytical grade and obtained from standard sources.

### 2.2 Plasmid construct

pRsSV‐GR was a gift from Dr Keith Yamamoto.^20^ pCEFL‐H_1_ receptor and TAT3‐Luc were previously generated in our laboratory.^21^, ^22^ IL6‐Luc was a kind gift from Prof. Dr Karolien De Bosscher (VIB Department of Medical Protein Research, University of Gent, Belgium).

### 2.3 Cell culture

HEK293T (human embryonic kidney stably transfected with SV40 T‐antigen) cells were obtained from the American Type Culture Collection (ATCC: Manassas, VA, USA) and cultured in DMEM supplemented with 10% fetal calf serum and 5‐μg/mL gentamicin. Cells were incubated at 37°C in humidified atmosphere containing 5% CO_2_. For cell passaging or plating, cells were first washed out with phosphate‐buffered saline (Invitrogen, Thermo Fisher Scientific) and then trypsinized using 1X trypsin‐EDTA.

### 2.4 Transfection and reporter gene assays

HEK293T cells seeded on 24‐well plates were cotransfected with the pRSV‐GR, pCEFL‐H_1_ receptor, and the luciferase reporter plasmids TAT3‐Luc or IL6‐Luc using the K2 Transfection System (Biontex, Munich, Germany) according to the manufacturer's instructions. After 4 hours, cells were seeded in 96‐well plates, and 24 hours later were starved overnight and then stimulated with ligands. After a kinetic assessment, luciferase activity was measured at the optimal time of 24 hours using the Steady‐Glo Luciferase Assay System according to the manufacturer's instructions (Promega Biosciences Inc San Luis Obispo) using a FlexStation 3 Multi‐Mode Microplate Reader (Molecular Devices). As shown before, no differences were observed in results normalized to Renilla‐Luc or to protein expression levels.^4^

### 2.5 Animals

Animal experiments were approved by the Institutional Committee for Use and Care of Laboratory Animals of the School of Pharmacy and Biochemistry of the University of Buenos Aires (CICUAL‐FFYB; EXP‐FYB N° 0040903/2015) and were performed in accordance with institutional and national guidelines for animal protection. Animal studies are reported in compliance with the ARRIVE guidelines.^23^, ^24^

Female BALB/c (H‐2^d^) mice were obtained from the animal facilities of the School of Veterinary Sciences (University of Buenos Aires, Argentina) and maintained in our animal facilities for use throughout these experiments. All animals were housed with four to five mice per cage and kept in the conditions of controlled light (12 h on, 12 h off), temperature, and humidity, with food and water ad libitum. Mice were used at the age of 6 to 8 weeks. Randomization was used to assign animals to different experimental groups and to collect and process data, with analysis performed by investigators blinded to the treatment groups.

### 2.6 Development of the experimental protocol

Thirty animals with an average weight of 20 gr were randomized and separated into two experimental groups. One group of 25 animals was sensitized with two intraperitoneal (ip) injections of 0.2‐mL PBS containing 20 μg of chicken egg white albumin (OVA) (grade V; Sigma Chemical Company) as the allergen and Aluminium hydroxide (2 mg) as the adjuvant 1 week apart. The other group of five animals was administered with 0.2‐mL PBS and remained as the naïve control group (N).

One week after the last injection, animals were exposed to aerosols of 5 mL of allergen OVA in PBS (3% (w/v)) during 20 min for 3 consecutive days. Aerosol exposure was performed within individual compartments of a mouse pie chamber using a nebulizer (SAN‐UP, Argentina, OVA solution flux 0.33 mL/min in air flux of 6 to 8 L/min). One hour after the last exposure, mice were intranasally treated with 7 μL of a water solution containing the different drugs. The experimental protocol is summarized as follows:

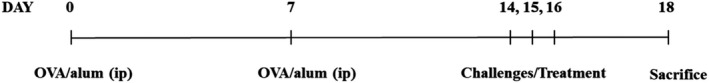

To perform the experiment, 30 animals were randomized and divided into six different experimental groups as follows:

1. Naïve group (N): negative control of the disease model. Animals not sensitized with OVA, only injected and airway challenged with PBS.
2. Asthmatic group (OVA): positive control of the disease model. Animals sensitized with OVA and intranasally treated with PBS.
3. Optimal dose of dexamethasone group (OD): positive control of the treatment. Animals sensitized with OVA and intranasally treated with a DEX solution in a dose corresponding to 1 mg/kg.^25^, ^26^, ^27^ This dose equals to 1.2 mg of inhaled corticosteroid in humans,^28^, ^29^, ^30^ in the order of recommended dose for the control of asthma symptoms according to GINA.^31^
4. Suboptimal dose of dexamethasone group (SD): Animals sensitized with OVA and intranasally treated with a 10‐fold dilution of OD solution (DEX solution in a dose corresponding to 0.1 mg/kg) expected to be inefficacious.
5. Azelastine group (AZE): Animals sensitized with OVA and intranasally treated with an AZE solution in a clinically equivalent dose corresponding to 0.5 mg/kg.
6. Cotreatment of suboptimal dose of dexamethasone and azelastine group (AD): Animals sensitized with OVA and intranasally treated with a solution containing DEX in a dose corresponding to 0.1 mg/kg and AZE in a clinically equivalent dose corresponding to 0.5 mg/kg.

It has been described that the allergen and the adjuvant in this particular strain promotes an antigen‐specific Th2 immune response, involving the induction of allergic parameters that reflect some pathological changes observed in bronchial asthma, such as high levels of allergen‐specific IgE, eosinophilic infiltrate in the airways and bronchial hyperreactivity.^32^ According to this, animals were euthanized and evaluated 48 hours after initiation of the treatment.

### 2.7 Pathologic analysis

Animals were euthanized with isoflurane. The chest wall was opened, and the animals were exsanguinated by cardiac puncture. Serum was prepared and stored at −20°C. The trachea was cannulated after blood collection. Bronchoalveolar lavage (BAL) was performed four times with 1 mL of sterile PBS. Lavage fluid was collected, centrifuged at 300*g* for 10 min, and the pellet was resuspended in 0.5‐mL PBS. BAL differential cell counts were performed on cytocentrifuge slides prepared by centrifugation of samples at 300*g* for 5 min (Cytospin 4; Shandon, Pittsburg, PA, USA). The slides were fixed and stained with a modified Wright‐Giemsa stain (Tincion 15, Biopur SRL, Rosario, Argentina), and a total of 200 cells were counted for each sample by optical microscopy. After lavage, one lung was extirpated and recollected in 1 mL of Quick‐Zol reagent (Kalium Technologies) for RNA extraction following the supplier's manual. The other lung was instilled with 10% buffered formalin, removed and fixed in the same solution. Following paraffin embedding, tissue sections for microscopy were stained with H&E or Periodic acid‐Schiff (PAS). An index of pathologic changes in H&E slides was obtained by examining 20 consecutive airways per slide at 400× magnification and scoring the inflammatory infiltrates around the airways and vessels for severity (0, normal; 1, ≤3 cells diameter thick; 2, 4 to 10 cells diameter thick; 3, ≥10 cells diameter thick). The Inflammatory Index was calculated by dividing the sum of the airway scores from each lung by the number of airways examined. A histological goblet cell score was obtained in PAS‐stained lung sections by examining 20 consecutive airways per slide at 400× magnification and categorized according to the abundance of PAS‐positive goblets (0, <5% goblet cells; 1, 5 to 25%; 2, 26 to 50%; 3, 51 to 75%; 4, >75%). The Mucus Index was calculated by dividing the sum of the airway scores from each lung by the number of airways examined for the histological goblet cell score.^33^

### 2.8 Assay of serum antibodies

ELISA plates (Nunc Maxisorp) were coated with OVA (10 μg/mL) in carbonate buffer (pH = 9.5) and placed at 4°C overnight. Mouse sera were diluted 1:2 x 10^4^ (IgE), 1:16 x 10^5^ (IgG1) and 1:200 (IgG2a). Biotinylated anti‐IgE mouse antibody (BD, Biosciences) or HRP‐conjugated goat anti‐mouse IgG1 or IgG2a (BD, Biosciences) were used as secondary antibodies. For IgE determination, streptavidin coupled to peroxidase enzyme (HRP, horseradish peroxidase‐streptavidin, Zymed, 1/4000) was added. Immune complexes were revealed with trimethylbenzidine substrate (TMB One‐Step; Dako, Carpenteria, CA, USA). Plates were read in a plate reader (Sunrise RC, Tecan) at 450 nm with λ correction at 570 nm after the addition of stop solution (H_2_SO^4^). Results are shown as optical density (OD) for a fixed dilution.^34^

### 2.9 RNA isolation & CDNA synthesis

Total cellular RNA was extracted using the Quick‐Zol reagent (Kalium Technologies, Buenos Aires, Argentina) following the supplier's manual. Total RNA was dissolved in RNase free water, denatured for 5 minutes at 65°C and RNA was quantified by spectrophotometric OD260 measurement using the Bioanalyzer (Agilent Technologies, Palo Alto, CA, USA). RNA samples were stored at −80°C until further use. One microgram of total RNA was used for cDNA synthesis, and in order to remove genomic DNA carryover, RNA samples were treated with 1.5 u of DNase I (Invitrogen) for 15 minutes at 25°C. Samples were then incubated at 65°C for 10 minutes following the addition of EDTA 25 nmol/L (Invitrogen, Thermo Fisher Scientific). Finally, they were reverse transcribed using the M‐MLV Reverse Transcriptase according to the manufacturer's instructions (Promega Biosciences Inc.). From each DNase I‐treated RNA sample, a nonreverse transcribed (‐RT) sample was similarly generated (reverse transcriptase was replaced with water). cDNA as well as ‐RT samples were kept at −20°C.

### 2.10 Quantitative polymerase chain reaction (QPCR)

Forward and reverse primer pairs were generated using the primer3Input online software (https://primer3plus.com/primer3web/primer3web_input.htm) and designs were based on publicly available mouse mRNA sequences. Primers were designed to have approximately 50% G/C content and to generate 75‐150‐bp amplicons. Primer pair specificity against target sequence was checked in the NCBI Genbank database using Primer‐BLAST (http://www.ncbi.nlm.nih.gov/). The sequences of the primers used to detect ArgI, IL‐4, IL‐5, and Muc5AC were designed by us and were as follows: ArgI forward 5’‐ CGTGTACATTGGCTTGCGAG ‐3’; ArgI reverse 5’‐ GCCAATCCCCAGCTTGTCTA ‐3’; IL‐4 forward 5’‐ ATGGATGTGCCAAACGTCCT ‐3’; IL‐4 reverse 5’‐ AAGCACCTTGGAAGCCCTAC ‐3’; IL‐5 forward 5’‐ AGGCTTTGTGCATGTTACCAAC ‐3’; Muc5AC forward 5’‐ GCAACTCCACCACCCCTACA ‐3’; Muc5AC reverse 5’‐ CCTTGCTTGAGGCCCCTGA ‐3’. Specific mRNA levels were normalized to CiclophylinA gene expression as recommended^35^ using the following primers also designed by us: CiclophylinA forward 5’‐ AGCACTGGAGAGAAAGGATTTG ‐3’; CiclophylinA reverse 5’‐ CCA GTGCCATTATGGCGTGT ‐3’. In all cases, primers were supplied by Genbiotech (Buenos Aires, Argentina) dissolved in water according to the supplier's instructions and kept at −20°C until use. qPCR monitoring and analysis was performed using the HOT FIREPol EvaGreen qPCR Mix Plus (Solis Biodyne, Tartu, Estonia). PCR reactions were performed in a total volume of 15 μL containing 3 μL of HOT FIREPol EvaGreen qPCR Mix Plus, 1.5 μL of a 10‐fold diluted cDNA and 0.375 μL of each forward (5 pmol/μL) and reverse primer (5 pmol/μL). Cycling conditions were a single preincubation step at 95°C for 10 minutes followed by 45 cycles of 30 seconds at 95°C, 30 seconds at 60°C, and 30 seconds at 72°C. To verify that the primer pairs used yielded single PCR products, a dissociation protocol was added after thermocycling, determining dissociation of the PCR products from 65°C to 95°C for 15 seconds. Finally, a cooling step was set for 20 seconds at 40°C.

To estimate the efficiency of the amplification reaction, serial half logarithm unit dilutions of cDNA were used and standard curves were generated. The linear slope of the standard curve for each primer pair was estimated using GraphPad Prism 6 software and the efficiency was calculated based on this following formula (1).

(1) $$\text{Efficiency}=10^{-(1/\text{slope})}$$

Additionally, the ‐RT samples and a water template were included in the analysis to confirm the absence of any residual DNA or contamination. All cDNA samples were analyzed in triplicates. Finally, the following formula (2) was used to calculate the fold induction of gene expression.

(2) $$\begin{array}{cc} & \text{Fold induction}\\ & \;=\frac{{\text{Efficiency of target gene}}^{\Delta \text{cp target}\;(\text{control}-\text{experimental group})}}{{\text{Efficiency of reference gene}}^{\Delta \text{cp reference}\;(\text{control}-\text{experimental group})}} \end{array}$$

### 2.11 Compliance with design and statistical analysis requirements

All animal groups have n = 5. Samples obtained from each animal were measured three times to test precision. Randomization was used to assign animals to different experimental groups and to collect and process data, with analysis performed by investigators blinded to the treatment groups.

The data and statistical analysis comply with the recommendations on experimental design and analysis in pharmacology.^36^ Graphs and statistical analysis were performed using GraphPad Prism 6.0 (GraphPad Software for Science). Data are presented as mean ± standard deviation (SD). For multigroup comparisons, a one‐way ANOVA test with a Tukey's posttest was performed using the same software package. No statistical differences between variances were observed along the whole work according to the Brown‐Forsythe test. *Post hoc* tests were run only if an overall statistically significant difference between the means were obtained. Statistical significance was accepted when *P* < .05.

## 3 RESULTS

### 3.1 AZE enhances DEX response in vitro

**Figure 1:**
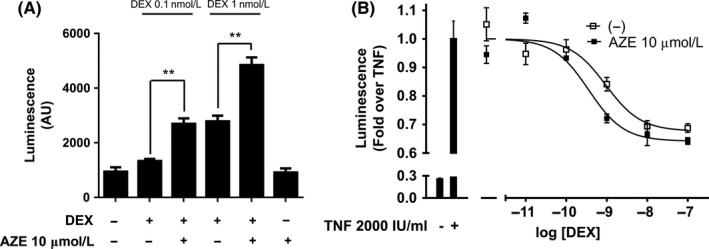
Azelastine enhances dexamethasone‐induced GR activity in vitro*.* (A) HEK293T cells cotransfected with the reporter TAT3‐Luc, H_1_ receptor, and GR coding plasmids were treated for 10 min with 10‐μmol/L azelastine (AZE) or not, as indicated, and incubated with 0.1 ‐nmol/L or 1‐nmol/L dexamethasone (DEX). (B) HEK293T cells were cotransfected with IL6‐Luc, H_1_ receptor, and GR coding plasmids and were pretreated with 10‐μmol/L azelastine (AZE) for 10 min and then with increasing concentrations of dexamethasone (DEX) for 4 h and finally treated with 2000 IU/mL TNFα for 18 h. Luciferase activity was determined as described in the methods section. Results are mean ± SD of five independent experiments performed in triplicates. *P < .01

We first validated that AZE had similar effect on DEX‐induced GR transcriptional activity in vitro*,* as previously reported for the antihistamines mepyramine and triprolidine.^4^ HEK293T cells were cotransfected with a luciferase encoding plasmid under the control of a synthetic promoter regulated by the GR (TAT3‐Luc) in combination with plasmids encoding for GR and H_1_ receptor. In this system we found that, while 10‐μmol/L AZE alone had no effect, pretreatment with 10‐μmol/L AZE enhanced 0.1 and 1‐nmol/L DEX‐induced luciferase activity by approximately 2‐fold (1331 ± 133 vs 2692 ± 348 and 2784 ± 360 vs 4842 ± 483, respectively) (Figure 1A). To evaluate if there was a potentiation of GR transrepression, we used the same cell system but with a different luciferase reporter. Cells were cotransfected with an IL‐6 promoter‐driven luciferase encoding plasmid, whose activity was induced pretreating the cells with 2000 IU/mL TNF‐α. Pretreatment with 10‐μmol/L AZE potentiated DEX‐induced repression of the IL‐6 promoter inducing a left shift in the dose‐response curve changing the pEC50 from 9.02 ± 0.2 to 9.45 ± 0.2 (Figure 1B).

### 3.2 AZE and DEX cotreatment reduces IgG and IgE

**Figure 2:**
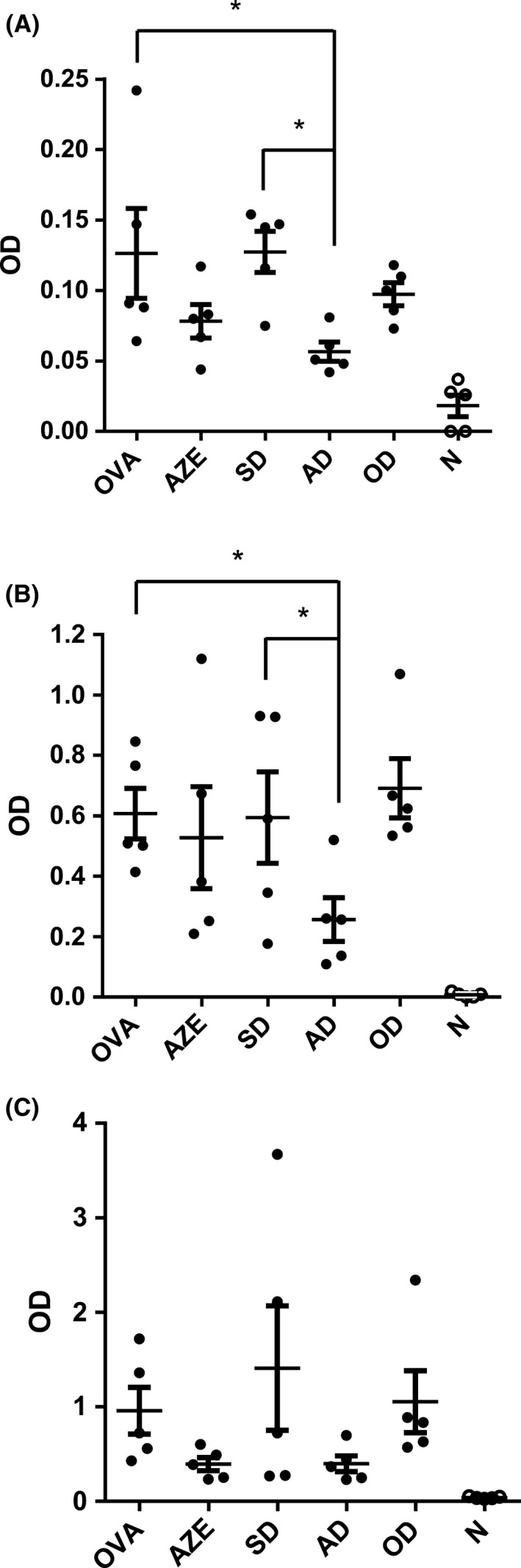
Effect of cotreatment on allergen‐specific humoral response. Serum levels of OVA‐specific (A) IgE, (B) IgG1, and (C) IgG2a antibodies were quantified in all experimental groups. N: Naïve animals; OVA: animals sensitized and treated with vehicle; AZE: animals sensitized with OVA and intranasally treated with azelastine; SD: animals sensitized with OVA and intranasally treated with a 10‐fold dilution of optimal dexamethasone solution. OD: animals sensitized with OVA and intranasally treated with an optimal dose of dexamethasone. AD: animals sensitized with OVA and intranasally treated with a solution containing azelastine and a suboptimal dose of dexamethasone. Dots represent the individual values obtained and horizontal lines represent the mean ± SD. *P < .05

As it was mentioned, atopic or allergic asthma is the most prevalent form of the disease and it is characterized by the presence of hypersensitivity reactions mediated by allergen‐specific IgE antibodies. We therefore analyzed the effects of the cotreatment on the serum levels of OVA‐specific IgG1, IgG2a, and IgE of the different experimental groups. Mice sensitized with OVA showed an increase in the specific immunoglobulin levels, which were significantly reduced by the cotreatment with 0.1‐mg/kg DEX and 0.5‐mg/kg AZE for IgE and IgG1. Remarkably, none of the treatments had an effect by themselves, suggesting a higher effectivity of the cotreatment (Figure 2A, 2, and 2). In this regard, it is worth mention that differently from what was previously reported, the optimal dose of DEX did not significantly reduced IgE levels.

### 3.3 AZE and DEX cotreatment reduces eosinophilia in BAL

**Figure 3:**
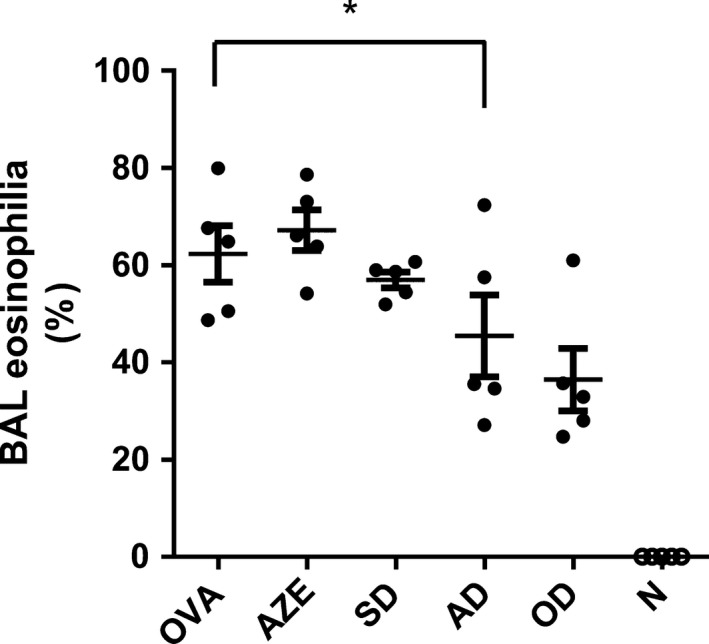
Effect of cotreatment on eosinophilia in bronchoalveolar lavage (BAL). BAL differential cell counts were performed on cytocentrifuge slides, fixed and stained with a modified Wright‐Giemsa stain. N: Naïve animals; OVA: animals sensitized and treated with vehicle; AZE: animals sensitized with OVA and intranasally treated with azelastine; SD: animals sensitized with OVA and intranasally treated with a 10‐fold dilution of optimal dexamethasone solution. OD: animals sensitized with OVA and intranasally treated with an optimal dose of dexamethasone. AD: animals sensitized with OVA and intranasally treated with a solution containing azelastine and a suboptimal dose of dexamethasone. Dots represent the individual values obtained and horizontal lines represent the mean ± SD. *P < .05

Accumulation of eosinophils in alveoli is a hallmark of allergic asthma and inflammation of the airways mediated by these cells is characteristic of the experimental model used herein.^32^, ^33^ We performed a differential count BAL cells of the different animals. Analysis showed that there is a rise in eosinophilia in OVA‐sensitized mice, confirming the induction of a pathological condition. Treatment with 1 mg/kg of DEX reduced it to almost the half (from 62.3 ± 5.8% to 36.5 ± 6.4%), while the administration of 0.1‐mg/kg DEX or AZE alone resulted ineffective. On the other hand, cotreatment with 0.5 ‐mg/kg AZE and 0.1‐mg/kg DEX resulted in one‐third decrease in the percentage of eosinophils (from 62.3 ± 5.8% to 45.4 ± 8.4%), (Figure 3). The number of other leukocyte subsets (macrophages, lymphocytes, and PMN) was not modified by any treatment (data not shown). These results show that only the higher dose of DEX as well as the lower dose of DEX in combination with AZE were effective in reducing the accumulation of eosinophils in mice airways. As it was observed for serum immunoglobulins, neither AZE nor 0.1‐mg/kg DEX alone reduced BAL eosinophilia. This result again suggests that the addition of AZE to a low dose of DEX could allow reaching a greater or equal therapeutic efficacy as that of higher DEX dose.

### 3.4 AZE and DEX cotreatment improves lung histopathology

**Figure 4:**
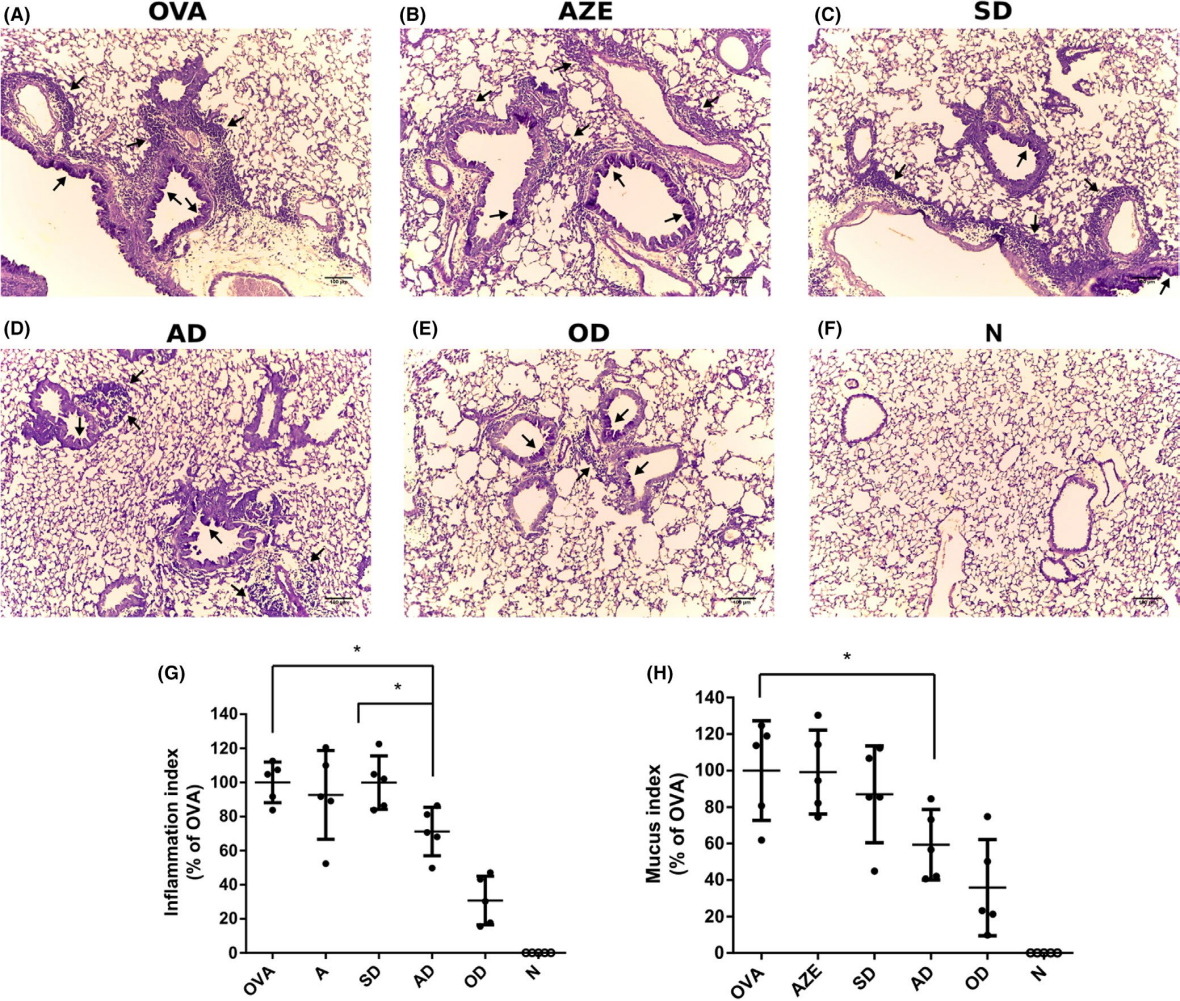
Effect of cotreatment on lung histopathology. (A‐F) Animal lungs treated as indicated were instilled and fixed with 10% buffered formalin. Following paraffin embedding, sections for microscopy were stained with Hematoxylin and PAS. Original magnification 400X. Black arrows point to prominent goblet cell hyperplasia and infiltration around airways and vessels. (G) An index of pathologic changes in H&E slides was obtained by scoring the inflammatory infiltrate around the airways and vessels for greatest severity. The Inflammation Index was calculated as the average of the airways’ index. (H) A histological goblet cell score was obtained in Periodic acid‐Schiff (PAS)‐stained lung sections by examining 20 consecutive airways from all groups of mice and categorized according to the abundance of PAS‐positive goblet. The Mucus Index was calculated as the average of the airways’ score. N: Naïve animals; OVA: Animals sensitized and treated with vehicle; AZE: animals sensitized with OVA and intranasally treated with azelastine; SD: animals sensitized with OVA and intranasally treated with a 10‐fold dilution of optimal dexamethasone solution. OD: animals sensitized with OVA and intranasally treated with an optimal dose of dexamethasone. AD: animals sensitized with OVA and intranasally treated with a solution containing azelastine and a suboptimal dose of dexamethasone. Dots represent the individual values obtained and horizontal lines represent the mean ± SD. *P < .05

Aerosol exposure of the animals to the allergen OVA produces an allergic‐inflammatory reaction of the airways that is reflected in pathophysiological changes in lung tissue which is typical of the clinical phenotype of all types of asthma. To evaluate whether suppressed BAL eosinophilia correlated with reduced lung pathology, we analyzed H&E and PAS lung‐stained sections of the different animals. Mice sensitized with OVA (Figure 4A) showed typical pathological changes of pulmonary allergic inflammation: eosinophils and mononuclear cell infiltration around airway and vessels, goblet cell hyperplasia and mucus production, which was not seen in the naïve group (Figure 4F). Animals who received an 1‐mg/kg DEX showed a small inflammatory infiltrate with reduced mucus production and no goblet hyperplasia (Figure 4E) while no significant differences in peribronchial and perivascular infiltrates were observed in animals which received 0.1‐mg/kg DEX or 0.5‐mg/kg AZE alone (Figure 4B,4 respectively) compared to allergic mice. On the other hand, animals cotreated with a 0.1‐mg/kg DEX and 0.5‐mg/kg AZE showed reduced peribronchial and perivascular infiltration and mucus production (Figure 4D) which resulted significantly different from the treatments alone. Results of semiquantitative histological scoring showed that 1‐mg/kg DEX induced a 70% of suppression of the allergic infiltration (from 100.0 ± 5.3% to 30.7 ± 6.4%), while the cotreatment with a 0.1‐mg/kg DEX and 0.5‐mg/kg AZE reduced it by 30% (from 100.0 ± 5.3% to 71.2 ± 6.3%). Treatments with the individual drugs did not significantly reduce this parameter (Figure 4G). Similar observations were made for PAS‐stained lungs sections in which the histological goblet cell score was reduced by 64% in animals who received a 1‐mg/kg DEX (from 100.0 ± 12.2% to 35.9 ± 11.8%); by 40% in those who received the cotreatment of 0.1‐mg/kg DEX and 0.5‐mg/kg AZE (from 100.0 ± 5.3% to 59.4 ± 8.6%); while no significant differences were observed in animals who received the treatments alone with respect to vehicle (Figure 4H), supporting the qualitative changes described above.

### 3.5 AZE and DEX cotreatment reduces the expression of asthma‐inflammatory genes

**Figure 5:**
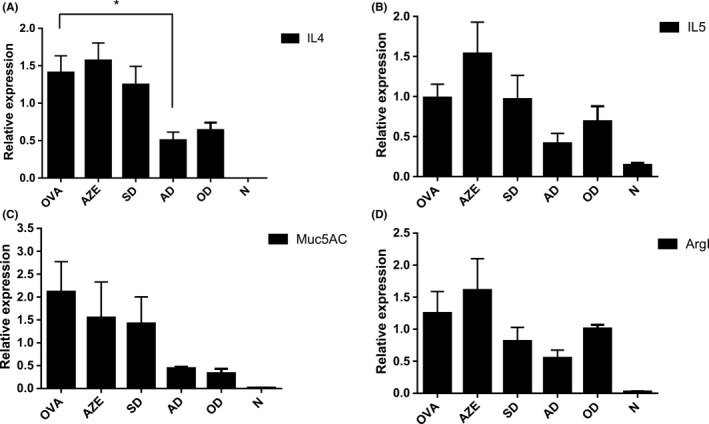
Effect of cotreatment on asthma‐inflammatory genes expression. Transcriptional response of four asthma‐inflammatory genes (A) IL‐4, (B) IL‐5, (C) Muc5AC, and (D) ArgI from lungs of all experimental mice. mRNA levels were quantified by qPCR as described in the methods section. N: Naïve animals; OVA: animals sensitized and treated with vehicle; AZE: animals sensitized with OVA and intranasally treated with azelastine; SD: animals sensitized with OVA and intranasally treated with a 10‐fold dilution of optimal dexamethasone solution. OD: animals sensitized with OVA and intranasally treated with an optimal dose of dexamethasone. AD: animals sensitized with OVA and intranasally treated with a solution containing azelastine and a suboptimal dose of dexamethasone. Results are mean ± SD. *P < .05

One key feature of this model is the development of an antigen‐specific Th2 immune response. Th2 cells are characterized by the release of specific cytokines, notably interleukin 4 (IL‐4) and interleukin 5 (IL‐5) that promotes airway inflammation rich in eosinophils, which are considered responsible for the asthmatic response (Mullane et al, 2014). Accordingly, we decided to analyze the expression of these cytokines in lung samples obtained from animals of all experimental groups. Furthermore, we also analyzed the expression of a mucin glycoprotein produced by epithelial cells (Muc5AC), to determine its correlation with the PAS results, as well as the Arginase I (ArgI), previously implicated in the pathogenesis of allergic airway disease and increased in BAL fluid from human asthmatic patients (Li et al, 2006; Morris et al, 2004; Zimmermann et al, 2003). Our results show that cotreatment with 0.1‐mg/kg DEX and 0.5‐mg/kg AZE induced a reduction in the expression of all the genes tested, which was statistically significant only for IL4, as compared to the treatments alone (Figure 5A‐D).

## 4 DISCUSSION

We show that treatment with a combination of AZE and a low dose of DEX can improve allergic lung inflammation in a well‐characterized model of experimental asthma. Specifically, we have shown a significant decrease in eosinophils in BAL lavage, fewer peribronchial and perivascular infiltrates and mucin‐producing cells. In addition, a diminished level of OVA‐specific IgE and IgG1 was detected.

GCs are among the most effective therapy to treat asthma and are usually the therapeutic choice for adults and children with severe asthma. International guidelines such as GINA (Global Initiative for Asthma) and NAEPP (National Asthma Education and Prevention) recommend GCs inhalation as therapy of choice to control long‐term persistent asthma, due to its anti‐inflammatory effects.^31^, ^37^ Suppression of proinflammatory genes reflect the molecular effects that result in an improvement of symptoms and pulmonary function, as well as a reduction of exacerbations and airways hyper response.^38^ However, there are at least two major issues concerning GCs’ therapy. On the one hand, there are GCs‐resistant patient populations which may represent 5%‐10% of the total.^39^ On the other hand, chronic treatment with a high dose of GCs could lead to the development of important adverse effects.^40^, ^41^, ^42^, ^43^

Concerning the improvement of GCs’ beneficial/adverse effects ratio, different strategies have been investigated, from chemical optimization of physiological corticoids to the development of Selective Glucocorticoid Receptor Agonists and Modulators (SEGRAs and SEGRMs). Complementary therapies are an alternative to control adverse effects, in which the addition of a different drug allows to reduce the dose of the corticoid. β2‐adrenoceptor agonists, theophylline, and antileukotrienes are widely used with this purpose, being the first ones, the most effective.^9^ Combination of β2‐agonists and corticoids is supported by scientific rationale. There are complementary effects on the physiopathology of asthma and synergic or at least additives effects at the molecular level. It has been reported that β2‐agonists increase GC‐induced GR nuclear localization and transcriptional activity.^44^

The efficacy of antihistamines for the treatment of asthma has been intensively studied over the last 50 years. Since mast cells were identified as key players in allergic asthma development, first and second generation of antihistamines were evaluated. Due to its proinflammatory effects histamine has been related to asthma development and abundant preclinical observations support this relationship.^12^ However, antihistamines have failed to be clinically effective for the management of asthma due to limited efficacy observed in several studies.^13^

We have previously reported a crosstalk mechanism between H_1_ receptor and GR‐mediated signaling pathways. This mechanism involves a dual regulation of GR activity by the H1R: a potentiation mediated by G‐protein βγ subunits and a parallel inhibitory effect mediated by a Gαq‐PLC pathway. Activation of the H_1_ receptor by its full agonists resulted in a composite potentiating effect. Intriguingly, inactivation of the Gαq‐PLC pathway by H_1_ receptor inverse agonists (ie, mepyramine and triprolidine) resulted also in a potentiation of GR activity.^4^ This was observed in heterologous expression systems, through gene‐reporter assays and measuring the expression of endogenous inflammatory‐related genes in physio‐pathological cell models.^4^ Now we show that another inverse agonist, AZE can also potentiate DEX‐induced GR activity in vitro (Figure 1). It can be inferred that if the molecular mechanisms underpinning this pharmacological potentiation observed in vitro would also exist in vivo in a clinically relevant target organ, cotreatment with DEX and AZE could present a therapeutic advantage over the treatment with each drug alone. In agreement with this hypothesis, our results show that the coadministration of AZE with a low dose of DEX, which had little or no effect by itself, improved DEX’s beneficial effects in an animal model of asthma. Our results support the clinical repurposing of antihistamines as GCs’ coadjudvants in asthma treatment.^45^

GC’s side effects are a crucial point in any pharmacological assessment. GCs are the most effective anti‐inflammatory drugs but its use as part of a treatment is limited by the existence of important adverse effects, such as osteoporosis, dyslipidaemias, body fat redistribution, muscular weakness and atrophy, insulin resistance, glucose intolerance and even diabetes.^46^ Given that anti‐inflammatory and adverse effects share the same molecular mechanisms and, as we described, antihistamines could enhance DEX‐induced GR activity both for gene transactivation and transrepression, it should be critical to address the potential modulation of GR adverse effects by antihistamines in order to assure their complete safety. Induction of adverse effects by long‐term use of corticoids at high doses might be reduced by diminishing corticoid dosage. However, corticoids’ adverse effects could be also enhanced by antihistamines.

There are some limitations to this study that warrant discussion. First, it remains to be confirmed whether the diminished lung allergy inflammation also correlates with lower airway hyperresponsiveness observed in asthma. Other shortcomings are related to the asthma protocol. The murine model used herein involves systemic sensitization with OVA as allergen followed by an aerosol challenge. This model reproduces many key features of clinical asthma such as elevated levels of allergen‐specific IgE, airway inflammation, goblet cell hyperplasia, epithelial hypertrophy, and airways hyperresponse^47^ and represents the most used preclinical model that resulted very useful to elucidate different aspects of the pathology of the disease and to search for new therapeutic treatments. However, it is an acute model whose suitability is limited due to minimal airway remodeling and the transient nature of the bronchial hyperreactivity and the eosinophilic inflammation that resolves within a few weeks.^32^ This settles the need of evaluating our hypothesis in a chronic model, which reproduces better the changes observed in patients,^48^ although they are still limited and there has not yet been widely adopted a single model. It is clear that although murine models do not exactly reproduce the pathophysiology of asthma, they have proved to be a valuable tool for investigation.^32^

In conclusion, our results contribute to the ongoing reevaluation of the use of antihistamines as add‐on drugs in GCs‐mediated anti‐inflammatory therapies. Based on the described crosstalk between H_1_ receptor signaling and GR activity, antihistamines could be repositioned as adjuvants for GC‐based therapies to diminish their adverse effects.^45^ Considering the widespread use of both types of drugs, the repositioning of antihistamines may make available new and safer therapeutic strategies, offering new options to improve the management of the symptoms in patients with asthma and other related inflammation‐related respiratory conditions.

## DISCLOSURE

The authors declare that the research was conducted in the absence of any commercial or financial relationships that could be construed as a potential conflict of interest.

## AUTHOR CONTRIBUTIONS

CDZ, AS, AG, and FM have made substantial contributions to conception and design of the work. CDZ, AS, GGG, IF, NF, CAD CS, AG, and FM have made substantial contributions to the acquisition of data, their analysis, and their interpretation. CDZ, AS, AG, and FM have been involved in drafting the manuscript. IF and CPF revised the manuscript critically for important intellectual content. All authors have given final approval of the version to be published.

## DATA AVAILABILITY STATEMENT

The data that support the findings of this study are available from the corresponding author upon reasonable request.
